# Genetic diversity and relationship between cultivated, weedy and wild rye species as revealed by chloroplast and mitochondrial DNA non-coding regions analysis

**DOI:** 10.1371/journal.pone.0213023

**Published:** 2019-02-27

**Authors:** Lidia Skuza, Izabela Szućko, Ewa Filip, Tomasz Strzała

**Affiliations:** 1 Department of Molecular Biology and Cytology, The Institute for Research on Biodiversity, Faculty of Biology, University of Szczecin, Szczecin, Poland; 2 The Centre for Molecular Biology and Biotechnology, Faculty of Biology, University of Szczecin, Szczecin, Poland; 3 Department of Genetics, Faculty of Biology and Animal Science, Wrocław University of Environmental and Life Sciences, Wrocław, Poland; National Cheng Kung University, TAIWAN

## Abstract

The genus *Secale* is small but very diverse. Despite the high economic importance, phylogenetic relationships of rye species have not been fully determined, and they are extremely important for the process of breeding of new cultivars that can be enriched with functional traits derived from wild rye species. The study analyzed the degree of relationship of 35 accessions of the genus *Secale*, representing 13 most often distinguished species and subspecies, originating from various seed collections in the world, based on the analysis of non-coding regions of the chloroplast (cpDNA) and mitochondrial genome (mtDNA), widely used in phylogenetic and population plant studies, because of a higher rate of evolution than the coding regions. There was no clear genetic structure between different species and subspecies, which may indicated the introgression between these taxa. The obtained data confirmed that *S*. *vavilovii* was very similar to *S*. *cereale*, which confirmed the assumption that they might share a common ancestor. The results also confirmed the divergence of *S*. *sylvestre* from other species and subspecies of rye. Areas that may be useful molecular markers in studies on closely related species of the genus *Secale* were also indicated.

## Introduction

Rye is a difficult object of genetic and breeding studies. The reason is the open-pollination, self-incompatibility and the relationship between heterozygosity and productivity, which arises as a result of inter-chromosomal gene interactions [1]. Rye has the largest genome ~ 7.9 Gbp among all diploid Triticeae [2], which is in 90% consisting of repetitive sequences. The genus *Secale* is also very diverse–it includes annual, perennial, self-pollinating and open-pollinating species of various morphologies. The classification system of the American Germplasm Resources Information Network (GRIN, http://www.arsgrin.gov) currently includes four species to the genus *Secale*: annual *S*. *cereale* L., annual *S*. *sylvestre* Host and *S*. *vavilovii* Grossh and perennial *S*. *strictum* (Presl.) Presl. (syn. *S*. *montanum*) [3,4]. All species within the genus *Secale* are diploid with 14 chromosomes, they cross with each other easily, and crossbreeding results in partially fertile hybrids [5,6]. Only *Secale sylvestre* has different characteristics (low crossability with other species, the lowest amount of t-heterochromatin [5] and the smallest genome (7.23 pg)[7] and is probably the most distant species [5,8], as indicated by many research results [9–15]. Rye (*Secale cereale* L.) is also an important and rich source of valuable genes encoding, e.g., high protein content, resistance to diseases as well as morphological and biochemical traits for wheat and a synthetic wheat–rye hybrid triticale (× *Triticosecale* Wittmack) improvement.

Rye has an advantage over other cereals in temperate climates because of its excellent tolerance to low temperatures, high levels of soil alumina and its ability to produce acceptable grain yields under drought stress conditions [16]. Rye grows on sandy or acidic soils or on poorly prepared terrain. It is widely adapted, but grows best in cold temperature areas. The colder climate in Central and Eastern Europe, which are the main rye growing regions in the world (http://www.fao.org/faostat), favors the high frost tolerance of rye and its ability to grow on poor soils [17]. Unfortunately, despite the economic importance, phylogenetic relationships within the genus *Secale* remain unclear. The reason for this may be the postulated multi-paths in the evolution of rye [11]. There is therefore a need to search for different verification methods for the proposed classification system and phylogenetic relationships.

The differences in the classification of the genus *Secale* may result from the use of various experimental methods, as described in detail previously [18]. Establishing phylogenetic relationships is extremely important for the process of breeding new cultivars, which can be enriched with functional traits derived from wild rye species, e.g., resistance to downy mildew and brown rust, resistance to lodging and pre-harvest sprouting and cms [19,20]. Phylogenetic and population studies often use genetic information contained in chloroplast (cpDNA) and mitochondrial DNA (mtDNA), as they contain two necessary sets of genes in plants [21–23]. Firstly, they encode many key proteins for basic cell bioenergy processes. Secondly, they encode many components necessary for the proper expression of their own genes. Considering the essential importance of these two sets of organelle genes, one can expect them to change very slowly during evolution [21–23]. In addition, organelle DNA is a useful tool in the search for species-specific molecular markers. However, phylogenetic studies of the genus *Secale* were almost exclusively based on the analysis of the nuclear and chloroplast genomes [18,24–34]. So far, two works have been published that include mtDNA: Isik et al. [33] and Skuza et al. [34]. None of the studies included the analysis of highly-variable non-coding sequences in organelle genomes, which due to the rapid evolution and accumulation of a higher number of deletions/insertions or substitutions than coding regions are considered very good markers when testing at the intraspecific level [35].

Recent studies have successfully proven that some parts of chloroplast genomes were more effective in illuminating phylogeny of land plants than cpDNA sequences frequently used in phylogenetic studies [36–39]. It is then interesting whether the non-coding regions of the chloroplast (cpDNA) and mitochondrial genomes (mtDNA) will confirm this dependence, all the more so as the mitochondrial genome of rye has not been sequenced so far, and complete data only on the *S*. *cereale* chloroplast genome sequence has been published in 2014 [40].

The current work analyzed the relationship of 35 accessions of the genus *Secale*, representing 13 most often distinguished species and subspecies, originating from different seed collections in the world. Twelve universal primers were selected for analysis [41–44], amplifying the non-coding regions of the chloroplast (cpDNA) and mitochondrial genomes (mtDNA), broadly used in phylogenetic and plant population studies [21–23]. The use of such consensus primers that are homologous to most coding regions, but amplify variable non-coding regions are very useful for phylogenetic and population studies [43–45].

The research was aimed at understanding the genetic diversity within the genus *Secale*, as well as the possibility of revising existing taxonomic classifications.

## Materials and methods

The plant material consisted of 35 accessions of the genus *Secale*, 13 cultivated and non-cultivated species and subspecies of rye, obtained from several world collections (Center for Biological Diversity Conservation in Powsin–Warsaw, Poland; United States Department of Agriculture–Agricultural Research Service, USA; Nordic Genetic Resource Center, Sweden). The list of species, along with the accession numbers for each sample, is given in S1 Table.

### DNA extraction, PCR amplification, and DNA sequencing

Genomic DNA was extracted from coleoptiles (0.2 g per sample) of etiolated 6- to 7-day-old seedlings using a FastDNA Green SPIN Kit (DNAeasy Plant Mini Kit–Wizard Genomic DNA Promega), according to the manufacturer’s instructions. The isolated DNA was subjected to quantitative and qualitative evaluation by measuring the absorbance at 230, 260, and 280 nm using a NanoDrop 2000c spectrophotometer (Thermo Scientific, Madison, USA). DNA isolation was performed at least three times.

#### PCR amplification of chloroplast (cp) non-coding (intron) DNA region

For the amplification of the cpDNA 6 non-coding (intron) regions, genomic DNA was isolated as described previously and the concentration was adjusted to ~50 ng/μl. Primer sequence list (5′-3′) for the amplification of different cpDNA regions is provided in S2 Table. PCR amplification was performed three times in a 25-μl reaction mixture containing approximately 50–150 ng genomic DNA template, 2.0–3.0 mM MgCl_2_, 0.2–1.0 mM of each dNTP, 0.1–1 μM of each primer, 0.1 mg BSA/ml, and 1 U *Taq* DNA polymerase. Specifics of reaction conditions and components for the amplification of each region are given in S4 and S6 Tables.

#### PCR amplification of mitochondrial (mt) non-coding (intron) DNA region

For the amplification of the mtDNA 6 non-coding (intron) regions, genomic DNA was isolated as described previously and the concentration was adjusted to ~50 ng/μl. Primer sequence list (5′-3′) for the amplification of different cpDNA regions is given in S3 Table. PCR amplification was performed three times in a 25-μl reaction mixture containing approximately 75–250 ng genomic DNA template, 2.5–4.0 mM MgCl_2_, 0.1–0.2 mM of each dNTP, 0.2–0.55 μM of each primer, 0.05 mg BSA/ml, and 1–1.5 U *Taq* DNA polymerase. Specifics of reaction conditions and components for the amplification of each analyzed region are presented in S5 and S7 Tables.

#### Electrophoresis

The products were separated on a 1.5% (m/v) agarose gel in a 1× TBE buffer (89 mM Tris, 89 mM boric acid, 2 mM Na_2_EDTA, pH 8.3). Ethidium bromide was added to the gel to a final concentration of 0.1 μg/ml. Electrophoresis was carried out in a Sub-Cell Model 192 PowerPac HV electrophoresis system (Bio-Rad) in a 1×TBE buffer at 100 V for approximately 1.5 h. Gel images were captured with a Gel Doc XR system (Bio-Rad). The bands were scored and analyzed with the Quantity One software (Bio-Rad). The size of the products was determined by comparison with the DNA ladder (MassRuler DNA Ladder Mix, Fermentas).

#### Sequence and alignment for data analysis

The majority of PCR products were purified and directly sequenced at Macrogen (Seoul, Korea) and Genomed (Warsaw, Poland). Sequencing was performed two times in both forward and reverse directions. Pairwise alignments were made using the sequences obtained from the forward and reverse primers. Multiple sequence alignment was performed using ClustalW. Genetic diversity was estimated based on the identification of unique haplotypes using DnaSP 5.10. [46]. This software was also used to calculate haplotype (Hd) and nucleotide (*π*_n_). The sequences reported in this paper have been deposited in the NCBI Genbank nucleotide sequence database with the accession numbers MH893827-MH894176 (S1 Table).

### Phylogenetic analysis

To infer phylogenetic relationship between analyzed species, we used concatenated sequences of the chloroplast genome (cpDNA) (alignment length– 6362 bp) and mitochondrial sequences (mtDNA) (alignment length– 4139 bp) for 37 individuals (including two sequences used as an outgroup). The dataset was analyzed as a partition consisting of two separate types of sequences (cpDNA and mtDNA) to allow different substitution models for both kinds of data.

Both partitions were first aligned separately with the MUSCLE algorithm [47] implemented in the Seaview software [48]. Next, sequences were cleaved to obtain equal alignment blocks and concatenated together (cpDNA and mtDNA) in one file.

Phylogenetic analyses were performed using two approaches: maximum likelihood (ML) and Bayesian inference (BI). ML analyses were carried out with PhyML [49,50] and RaxML 7.7.1 [51,52]. In PhyML, smart model selection function [53] was used with the Akaike Information Criterion along with five random starting trees and SPR type tree improvement. The bootstrap analysis (1000 replications) was used to test tree topology. In RaxML, the GTR +I +G substitution model was used as the best-fit model selected using jModelTest 2.1.10 [54] for both partitions. Hundred replications were used in the bootstrap analysis to test tree topology. MrBayes 3.2.6 [55] was used for the Bayesian tree, with a separate mixed +I +G [56] model for each partition, and the analysis was run with two independent runs (four chains each) starting from random trees. Trees were sampled every 100^th^ generation of the Markov chain step for 25,000,000 generations with 25% burn-in. Final tree set consisted of trees probed when the average standard deviation between robots was stabilized significantly below 0.01.

All trees were visualized with FigTree [57] and compared with each other.

#### Distance between and within clades

We estimated distances between and within clades revealed on the tree, as we were interested in determining the relationships between species. The analyses were conducted using the 2-parameter Kimura model [58] with gamma distribution (shape parameter = 0.05) of the rate variation among sites and deletion of all positions with gaps, using MEGA X [59].

## Results

### Characteristics of cpDNA non-coding sequences

The size polymorphism of the PCR products obtained was not detected within the analyzed regions. Due to the difficulty in amplifying two regions *trnL* (UAA) 3' exon-*trnF* (GAA) and *trnK* [tRNA-Lys (UUU) exon 1] -*trnK* [tRNA-Lys (UUU) exon2], further analyses were based on the results obtained from the four regions (Table 1).

**Table 1:** Characterization of nucleotide variability of analyzed plastid regions (cpDNA) in the type of *Secale*.

Nucleotide variability	^a^Regions of cpDNA				Total	Mean
	1	2	3	4		
Length [bp]	815	600	653	963	3031	758
No. of Conserved sites [C]	671	248	490	889	3054	575
No. of Variable sites [V]	114	336	159	68	677	271
No. of Parsim-info [Pi]	7	31	59	42	139	81
No. of InDel site	17	16	19	29	81	32.40
Max. InDel size	14	1	2	1	-	2,61
No. of Substitutions sites	106	142	118	40	406	102
No. of Haplotype	8	12	5	16	41	8.40
Haplotype diversity [Hd]	0.408	0.565	0.341	0.765	-	0.520
Nucleotides diversity [Pi]	0.011	0.034	0.010	0.006	-	0.015

^**a**^Analyzed plastid regions (cpDNA): **1-***atpB-rbcL* intergenic spacer; **2**—*trnT*(UGU)-*trnL*(UAA)5’exon intergenic spacer; **3-** *trnL*(UAA) intron intergenic spacer; **4**- *trnD*[tRNA-Asp(GUC)]-*trnT*[tRNA-Thr(GGU)] intergenic spacer.

A total of 3,031 bp from 140 sequences were analyzed. The longest identified noncoding region in the chloroplast genome was *trnD* [tRNA-Asp (GUC)]-*trnT* [tRNA-Thr (GGU)]. Four regions: *atpB-rbcL*, *trnT*(UGU)-*trnL*(UAA)5' exon, *trnL*(UAA) intron and *trnD*[tRNA-Asp(GUC)]—*trnT*[tRNA-Thr(GGU)] showed differences on both intraspecific and interspecific levels. The most variable regions (*trnT* (UGU)-*trnL* (UAA) 5' exon and *trnD*[tRNA-Asp(GUC)]—*trnT*[tRNA-Thr(GGU)]) were selected, where the number of haplotypes was 12 and 16, respectively (Table 1).

Substitutions were the main source of variation, despite the fact that the studied regions also showed indels (Table 1). The number of identified indels ranged from 16 to 29 (*trnD*[tRNA-Asp(GUC)]-*trnT*[tRNA-Thr(GGU)]) (Table 1). The size of indels ranged from 1 bp in regions *trnD*[tRNA-Asp(GUC)]-*trnT*[tRNA-Thr(GGU)] and *trnT*(UGU)-*trnL*(UAA)5' exon to 14 bp in the *atpB-rbcL* region (Table 1).

On average, 8.4 haplotypes were identified in the four analyzed non-coding regions (Table 1). The highest number of haplotypes was determined in two regions: *trnD*[tRNA-Asp(GUC)]-*trnT*[tRNA-Thr(GGU)] (16) and *trnT*(UGU)-*trnL*(UAA)5'exon (12). The most frequently occurring haplotype was haplotype 1 in the non-coding *atpB-rbcL* region (S8 Table). The haplotypes diversity ranged from 0.341 to 0.765 in the *trnD*[tRNA-Asp(GUC)]-*trnT*[tRNA-Thr(GGU)] region, whereas the nucleotide diversity ranged from 0.006 to 0.034 in the *trnT*(UGU)-*trnL*(UAA)5' exon region.

**Fig 1:**
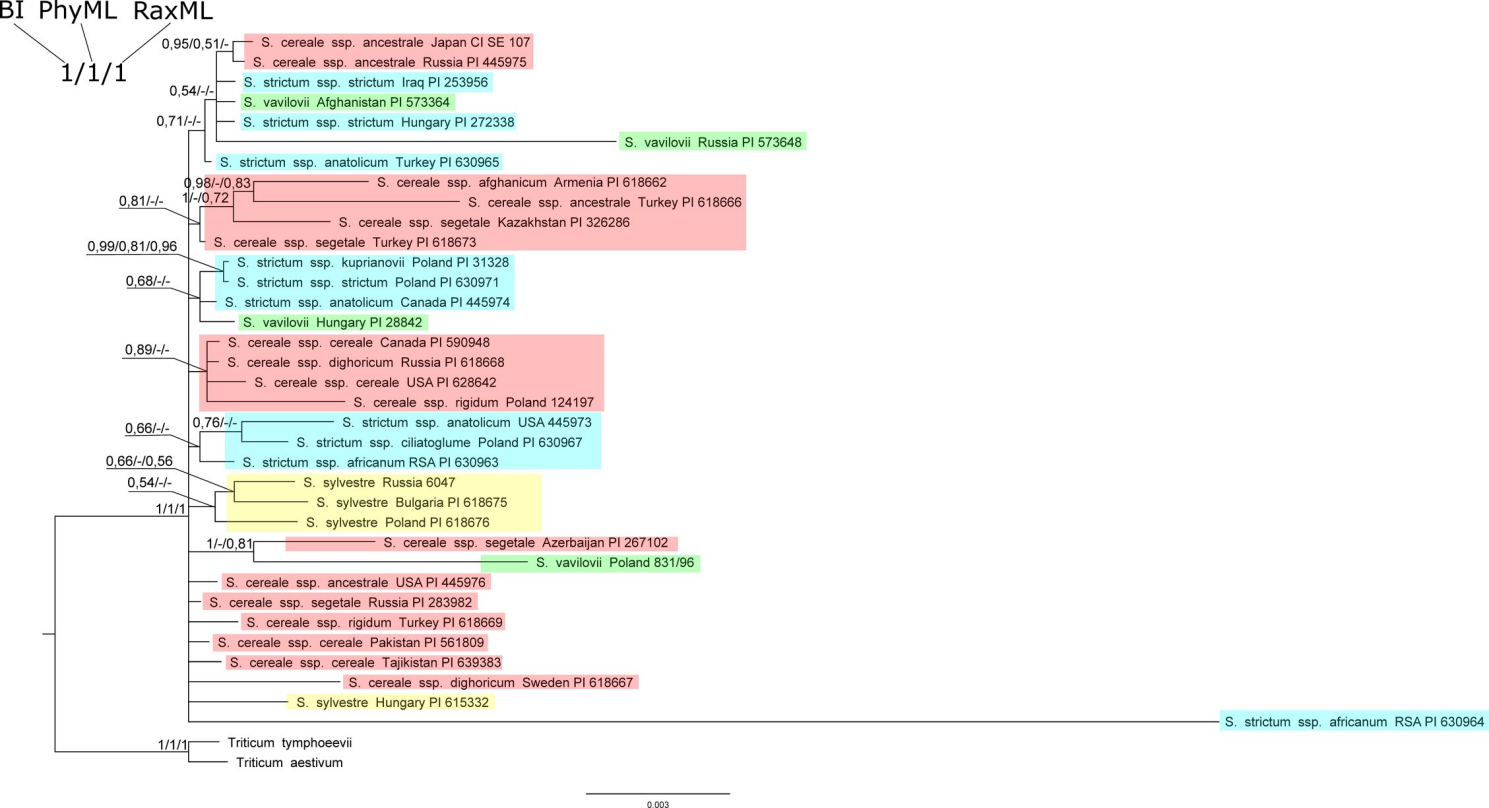
Bayesian tree based on the cpDNA noncoding sequences. Bootstrap values (RaxML and PhyML) and posterior probability values are presented along nodes (values below 0.5 are not presented).

The BI, PhyML, and RaxML trees of all analyzed non-coding sequences of chloroplast DNA presented a similar topology with minor differences (Fig 1). The tree was not very informative and the topology of the tree was mainly polytomous, and one node connected all the analyzed sequences.

A few subgroups could be noticed within this group, formed mainly by *S*. *strictum* and *S*. *sylvestre* species. *S*. *africanum* (RSA1) formed a separate group (Fig 1). *S*. *vavilovii* was dispersed within the entire second group; only *S*. *vavilovii* Afghanistan and Russia showed a relative similarity. S. *cereale* species also did not form groups consistent with the current classification.

Noteworthy are the results obtained for *S*. *strictum* ssp. *africanum*. Two samples originating from South Africa were analyzed. The results clearly suggested that the origin of these seeds was not homogeneous–the second sample (RSA1) formed a separate subgroup (Fig 1).

Sequence similarity, depending on the origin, was not consistent. Only *S*. *sylvestre* species (Poland, Bulgaria, Russia) formed a monophyletic clade.

### Characteristics of mtDNA non-coding sequences

Products were obtained for all analyzed mtDNA regions, as a result of the amplification (Table 2). The size polymorphism of the PCR products obtained was not detected within the analyzed regions.

**Table 2:** Characterization of nucleotide variability of analyzed mitochondrial regions (mtDNA) in the type of *Secale*.

Nucleotide variability	^a^Regions of mtDNA						Total	Mean
	1	2	3	4	5	6		
Length [bp]	1483	1642	626	134	120	166	4171	695
No. of Conserved sites [C]	1438	1088	626	63	120	166	3546	591
No. of Variable sites [V]	0	509	0	71	0	0	580	97
No. of Parsim-info [Pi]	0	106	0	71	0	0	177	30
No. of InDel site	0	40	0	5	0	0	45	8
Max. InDel size	0	5	0	1	0	0	-	4.5
No. of Substitutions sites	0	222	0	70	0	0	292	49
No. of Haplotype	1	12	1	2	1	1	18	3
Haplotype diversity [Hd]	0.000	0.867	0.000	0.329	0.000	0.000	-	0.199
Nucleotides diversity [Pi]	0.000	0.029	0.000	0.186	0.000	0.000	-	0.036

^**a**^Analyzed mitochondrial regions mtDNA: **1-** *nad1*exon B-*nad1*exon C intron intergenic spacer; **2**- *nad4*/1-2 intergenic spacer; **3**- *nad4L-orf25* intergenic spacer; **4**- *rps12-1*/*nad3-2* intergenic spacer; **5**- *rps12-2*/*nad3-1* intergenic spacer; **6**- *rrn5/rrn18-1* intergenic spacer.

A total of 4,171 bp from 210 sequences were analyzed. The longest identified non-coding region in the mitochondrial genome was: *nad4/1-2* (1642 bp) and it showed the highest polymorphism within the mtDNA noncoding regions of genus *Secale* (Table 2).

Two regions, *nad4/1-2* and *rps12-nad3(1)*, showed variation at both intraspecific and interspecific levels. The remaining regions were conserved.

The number of haplotypes in the variable *nad4/1-2* and *rps12-nad3*(2) regions was 12 and 2, respectively (Table 2). Although the studied regions also contained indels, substitutions were the main source of variation. The number of identified indels ranged from 0 to 40 (in *nad4/1-2*) (Table 2). The size of indels ranged from 1 bp in the *rps12-nad3*(2) regions to 5 bp in the *nad4/1-2* region (Table 2).

On average, 3 haplotypes were identified among the six analyzed sequences (Table 2). Most haplotypes (12) were detected in the *nad4/1-2* region. The most frequently occurring was haplotype 1 in the non-coding *rps12-nad3*(2) region (S9 Table). The haplotypes diversity ranged from 0 (conserved regions) to 0.867 in the *nad4/1-2* region, while the nucleotides diversity ranged from 0 to 0.186 in *rps12-nad3*(2) (Table 2).

**Fig 2:**
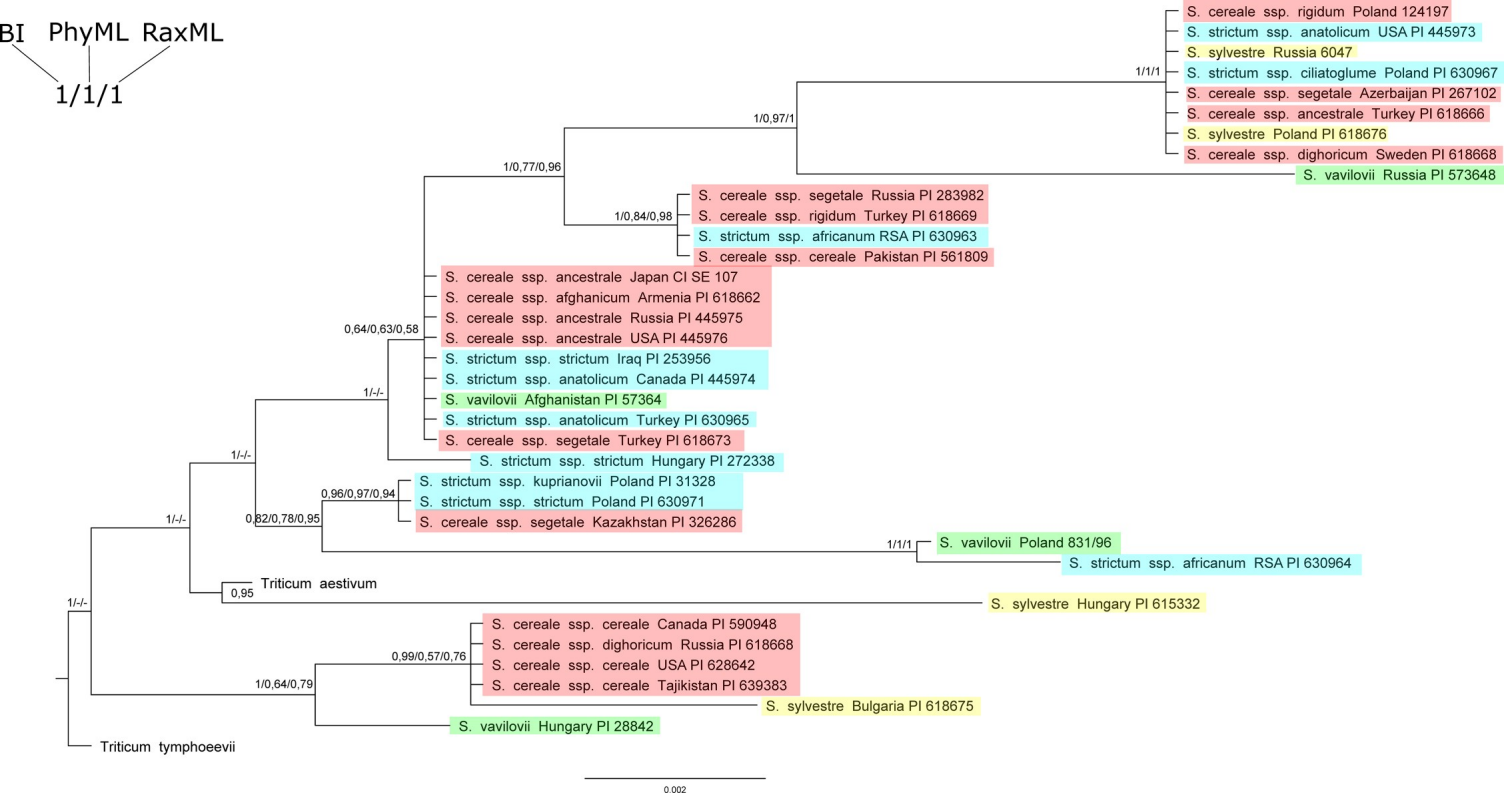
Bayesian tree based on the mpDNA noncoding sequences. Bootstrap values (RaxML and PhyML) and posterior probability values are presented along nodes (values below 0.5 are not presented).

The BI, PhyML, and RaxML trees of all analyzed non-coding sequences of mitochondrial DNA presented a similar topology with slight difference (Fig 2). The tree was polytomous, but only at the branch termini. The main nodes branched out dichotomously, and polytomy at the branch termini resulted from the fact that the sequences that formed particular clades were the same.

The obtained tree presents the division of rye species into two groups (Fig 2). A separate group consisted of the following species: *S*. *cereale* ssp. *cereale* (Canada, USA, Tajikistan), *S*. *cereale* ssp. *dighoricum* (Russia) forming a common subgroup with *S*. *sylvestre* (Bulgaria) and *S*. *vavilovii* (Hungary).

In the second group, *S*. *sylvestre* (Hungary) was the least similar to the others, followed by *S*. *vavilovii* (Poland) and *S*. *strictum* ssp *africanum* (RSA1), forming one subgroup and showing similarity to the following species subgroup: *S*. *strictum* ssp. *kuprijanovii* (Poland), *S*. *strictum* ssp. *strictum* (Poland) and *S*. *cereale* ssp. *segetale* (Kazakhstan).

There were no clear species and geographic specific patterns (e.g. monophyly) on the tree, as resolved clades were consisted of different species from different geographic locations. Two analyzed samples of *S*. *strictum* ssp. *africanum* were located in two distant similarity groups, as in the cpDNA analyses.

### The pooled analysis of sequences of non-coding regions of chloroplast and mitochondrial DNA–Phylogenetic analysis

**Fig 3:**
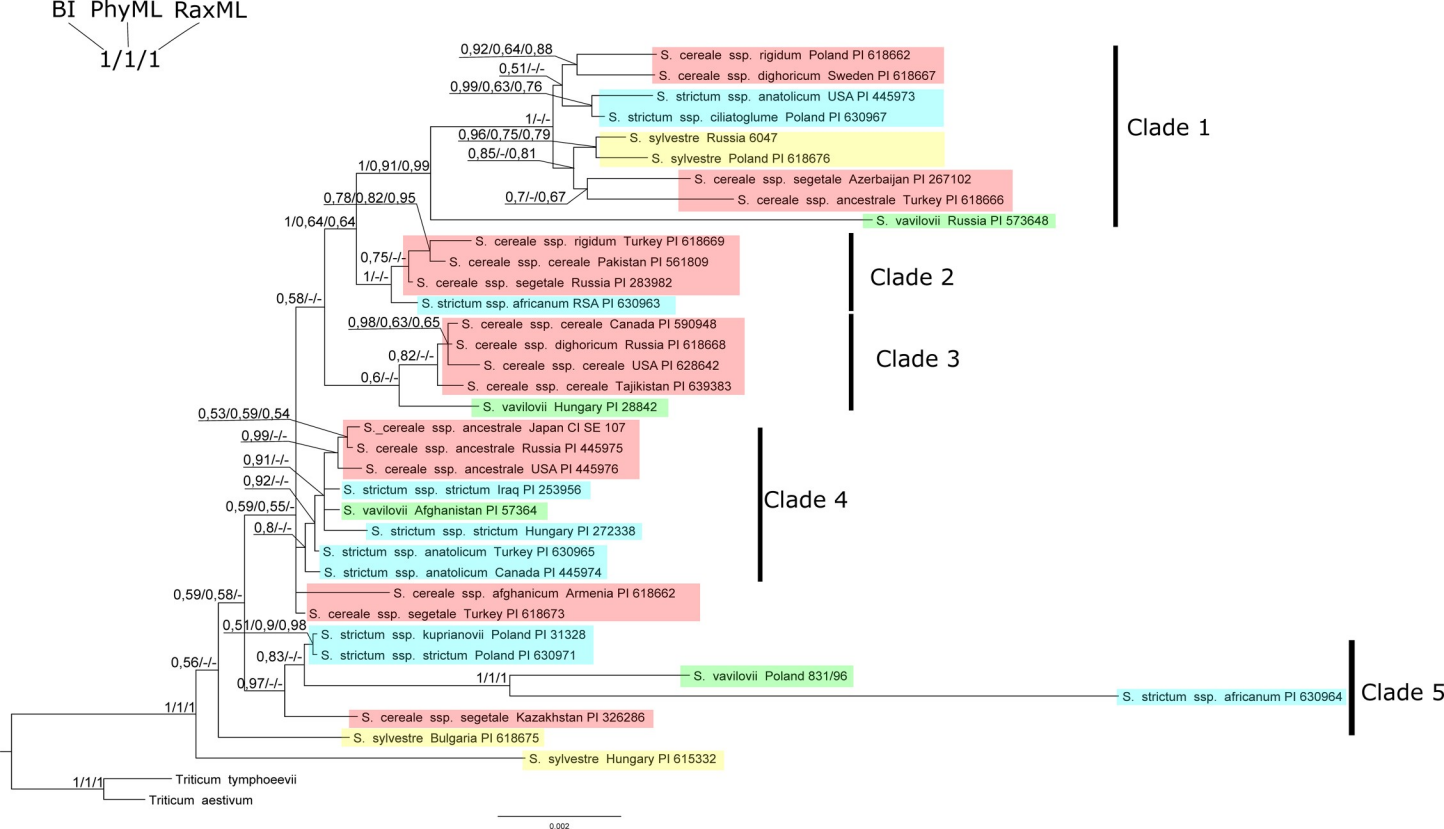
Bayesian phylogenetic tree of 35 *Secale sp*. individuals and two *Triticum sp*. representatives used as an outgroup. Bootstrap values (RaxML and PhyML) and posterior probability values are presented along nodes (values below 0.5 are not shown).

The BI, PhyML, and RaxML trees of all analyzed non-coding sequences of chloroplast and mitochondrial DNA presented a similar topology with slight differences (Fig 3). There were no or almost no clear systematic or geographical specific patterns (e.g. monophyly) on the tree, and the distance between representatives of some species was similar (or larger) than between representatives of different species. Lack of visible monophyletism suggested that the species division did not fully reflect the reality.

We were able to distinguish five distinct genetic clades on the tree that were used for distance analyses (Fig 3). The first clade consisted of 4 *S*. *cereale* subspecies, 2 subspecies of *S*. *strictum*, *S*. *sylvestre* (Russia and Poland) and *S*. *vavilovii* (Russia). The second clade was formed of three *S*. *cereale* and *S*. *strictum* ssp. *africanum* (RSA) subspecies. The third clade mainly grouped *S*. *cereale* and *S*. *vavilovii* subspecies (Hungary). The fourth clade included subspecies of *S*. *cereale*, *S*. *strictum* ssp. *strictum* (Iraq, Hungary), *S*. *s*. ssp. *anatolicum* (Turkey and Canada) and *S*. *vavilovii* (Afghanistan). The last highlighted clade contained *S*. *vavilovii* (Poland) and *S*. *s*. ssp. *africanum* (RSA1).

*S*. *sylvestre* (Hungary) and *S*. *sylvestre* (Bulgaria) formed a separate group. The other two accessions of this species (Russia and Poland) were similar, but they were in a separate clade 1. Most of the accessions of the cultivated species were located in clade 3, with the exception of *S*. *c*. ssp. *cereale* from Pakistan, which was located in clade 2 and showed a high similarity to *S*. *c*. ssp. *rigidum* from Turkey. *S*. *c*. ssp. *cereale* (Canada, Russia, USA) was also genetically similar to *S*. *c*. ssp. *dighoricum* (Russia).

Similarly, 3 accessions of *S*. *c*. ssp. *ancestrale* (Japan, Russia, USA) grouped together in clade 4, but showed similarity to *S*. *s*. ssp. *strictum* (Iraq, Hungary) and *S*. *vavilovii* (Afghanistan). The remaining *S*. *c*. ssp. *ancestrale* (Turkey) was similar to *S*. *c*. ssp. *segetale* (Azerbaijan) (clade 1). *S*. *vavilovii* was dispersed in four clades, but it was always distant from other species (Fig 3).

### Distance between and within clades

The average pairwise distance between sequences within clades was estimated at 0.003313 with a range from 0.000296 for clade 4 to 0.011245 for clade 5 (Table 3). On the other hand, the average distance between different clades was 0.0055 ranging from 0.00108, for clade 3-clade 4 distance, to 0.0126 for clade 1-clade 5 distance (Table 4). When compared, distances between clades were on average higher than within them. Nevertheless, the lowest distance within the clades was nearly four times lower than the same value between clades and the highest distance within clades was only slightly different than between them.

**Table 3:** Estimates of average evolutionary divergence over sequence pairs within clades. The number of base substitutions per site from averaging over all sequence pairs within each group are shown (MPD) along with standard error estimate(s) (SE).

	MPD	SE
Clade 1	0.003959	0.000301
Clade 2	0.000493	0.000157
Clade 3	0.00057	0.000173
Clade 4	0.000296	9.88E-05
Clade 5	0.011245	0.000853
**Average**	0.003313	0.000317

**Table 4:** Estimates of evolutionary divergence over sequence pairs between clades. The number of base substitutions per site from averaging over all sequence pairs between groups are shown. Standard error estimate(s) are shown above the diagonal.

**Clade 1**		0.0006099	0.0006728	0.0007307
**Clade 2**	0.0057124		0.0003744	0.0003348
**Clade 3**	0.0059956	0.0012996		0.0003018
**Clade 4**	0.0063539	0.0012154	0.0010806	
**Clade 5**	0.0126718	0.0073360	0.0071080	0.0062579

## Discussion

The genetic information contained in both chloroplast (cpDNA) and mitochondrial DNA (mtDNA) is often analyzed in phylogenetic and population studies. However, for most species, including rye, the data is incomplete. So far, rye mtDNA has not been fully sequenced, while in 2014, complete data on the *S*. *cereale* chloroplast genome sequence has been published [40]. Only in 2007, Isik et al. [33] analyzed the organelle genomes of S. *cereale* cultivars originated from different geographical regions using the PCR-RFLP method. The study used 7 cpDNA fragments and 4 mtDNA fragments for both coding and non-coding regions. Each amplified sequence was digested with 13 different restriction enzymes. The study of Isik et al. [33] proved that the mitochondrial genome, as compared to the chloroplast genome, showed a higher level of organelle polymorphism between analyzed rye cultivars. Our research [34], consisting in the analysis of mtDNA of seven species and subspecies of rye based on the RFLP method, showed the division of the analyzed taxa into two groups. The first included *Secale cereale* ssp. *segetale* and *Secale sylvestre*, and the second group comprised the remaining of the analyzed species (*Secale strictum*, *Secale strictum* ssp. *kuprijanovii* and *Secale vavilovii*, *Secale cereale* and *Secale strictum* ssp. *africanum*), which did not overlap with the existing classification system.

In 2016, Hagenblad et al. [25] analyzed the genetic diversity of 76 accessions of wild, feral and cultivated rye based on SNP polymorphisms. They performed an analysis of five chloroplast SSRs, derived from *Lolium* and wheat. Discriminant analysis of principal components (DAPC) of cpSSR data indicated very large genetic variation within the genus *Secale* and did not reflect taxonomic groups, except for *S*. *strictum* and *S*. *africanum*, which formed a separate cluster.

In the latest study on phylogenetic analysis of the genus *Secale* [24] the following collection of plants representing 11 species and subspecies of rye was studied using SSR and EST nuclear sequences. These results indicated high genetic diversity of S. *strictum* in comparison to other *Secale* species. The existence of two separate clusters of different species and subspecies was also found, and their division was not consistent with taxonomic affiliation, but was based on geographical origin (samples from Asia and outside of Asia). A clear separation between *S*. *sylvestre* and the rest of the genus was also revealed.

The analyses in the current study have included commonly used regions as well as those that are much less frequently applied in phylogenetic analyses, since no relationship analyses have been performed so far within the genus *Secale* based on non-coding sequences of organelle genomes. The amplification was carried out using universal primer pairs that allowed the amplification of non-coding regions dividing the two coding fragments in most plants [42,43,45]. The use of primers that are homologous to most coding regions, but amplify variable non-coding regions is very useful for phylogenetic and population studies [42,60,61].

### Evaluation of sequence variability of cpDNA non-coding regions in rye species

The cpDNA regions are widely used as markers in phylogenetic and phylogeographic studies, however, little is known about their usefulness for studying the relationships between closely related species, especially in monocotyledonous plants [61,62]. The slow rate of cpDNA-specific evolution hinders taxonomic analyses at lower levels, especially at the population level. In turn, non-coding regions are characterized by a higher rate of evolution than the coding regions–for example, the *trnL-trnF* region evolved 1.93–1.72 times faster than *rbcL* in certain genera of *Gramineae* [35].

The most commonly used non-coding regions, such as the *trnL*, *trnL-trnF* and *trnK* intron*/matK* have proved useful in phylogenetic analyses of some plant groups [63, 64], but often showed too low resolution in other groups, at least in some clades of angiosperms [65–67]. To obtain additional data and a better resolution for phylogenetic studies, sequences from these popular regions are often used in combination with other cpDNA sequences or with mtDNA and nDNA sequences [68–72], because additional information is often necessary to provide a satisfactory hypothesis regarding phylogenesis.

Studies on cpDNA clearly indicate that the utility in phylogenetic analyses of different cpDNA non-coding regions within a given taxonomic group can vary enormously [68, 72–77], and the selection of the appropriate cpDNA region is often difficult due to the lack of information about the rate of evolution between different non-coding cpDNA regions. This was confirmed by the results obtained in the present work, because they indicates a high variability of the studied regions of the chloroplast genome in the majority of taxa (Table 1). The most useful for the analysis of closely related taxa turned out to be *trnT* (UGU)-*trnL*(UAA)5'exon and *trnD*[tRNA-Asp(GUC)]-*trnT*[tRNA-Thr(GGU)] regions, especially when used in combination. These areas should serve as useful molecular markers for studies of closely related species, at least at inter-specific level in the genus *Secale*.

cpDNA shows a much more stable structure in case of intramolecular rearrangement than the mitochondrial genome of plants. However, the rate of plastid genome substitution is 3–4 times higher than that of plant mtDNA [78]. Most of the variability observed in cpDNA non-coding regions concerns insertion-deletion (indel) mutations, but, as stated by other authors, they should be treated with caution as they may indicate heteroplasmy [79]. However, indels were analyzed in the following studies due to the fact that they were found to be common and often phylogenetically informative [35,80,81]. In our studies the phylogenetic indeles context were not analysed because algorithms describing substitution models are not able to model indel-type changes and they are removed by tree-creating programs. A total of 81 indels have been identified–an average of 1.03 per sample (Table 1). This number is not very high compared to the results presented by other authors. Shaw et al. [82], analyzing 10 groups representing three different angiosperm lines: Atropa vs. Nicotiana (asteridy); Lotus vs. Medicago (rosids); and Saccharum vs. Oryza (monocotyledons), characterized a total of 1260 indels–an average of 3.0 per sample. It can be concluded that the appropriate combination of regions alongside the described indels as an additional factor, strengthens the usefulness of cpDNA for phylogenetic studies.

This study identified from 5 to 16 haplotypes per DNA fragment, and the most frequent was haplotype 1 in the non-coding *atpB-rbcL* region (S8 Table). The *atpB-rbcL* region, located between the gene encoding the large ribulose-1,5-bisphosphate carboxylase/oxygenase (*rbcL*) subunit and the gene encoding the β subunit of chloroplast ATP synthase (*atpB*) has different size in different taxonomic groups. According to a study conducted by Chiang [42], its size in the studied species ranged from 524 bp to about 1000 bp [42]. In *S*. *cereale*, it has a length of 774 bp [40]. The results obtained in the present work are comparable, since the size of this region in rye species is 815 bp (Table 1). In addition, it is characterized by a moderate variability. The number of haplotypes for this region is 8, which considering the 13 analyzed taxa is quite high. The occurrence of deletions and insertions as well as numerous nucleotide substitutions is a common phenomenon in the analyzed region. In addition, *atpB-rbcL* has been shown to be AT rich. The majority of non-coding regions rich in these base pairs show a low number of functions [83].

The next analyzed region–*trnT* (UGU)-*trnL* (UAA) 5' exon is located between the tRNA genes. It shows a high frequency of insertions or deletions, depending on the species, which makes it possible to use them as genetic markers. The sequence length of this region in the current work was 600 bp (Table 1), similarly in *S*. *cereale* it was 615 bp [40]. In general, this area was characterized by a variable length, from 298 bp to 700 bp, as demonstrated in studies in certain bryophytes, gymnosperms and angiosperms [44]. Exceptionally, its size in rice was 770 bp, in tobacco– 710 bp, and it was much shorter in *Marchantia polymorpha*– 188 bp [84]. This region proved to be a very good marker for studying closely related *Secale* species, due to its high variability.

The region of the *trnL*(UAA) intron is well known and its sequences have already been used to identify phylogenetic relationships between closely related species or to identify plants [85]. There is a view expressed repeatedly in the literature that among the regions of the chloroplast genome, the *trnL*(UAA) intron, belonging to type I introns, is less variable due to its catalytic properties and secondary structure, and therefore, may be more useful at higher taxonomic levels [44, 85–88]. This was confirmed by the results obtained in the presented work. The *trnL*(UAA) intron size in most of the species studied was from 254–767 bp. It was of medium size (594 bp) in S. *cereale* [40], and the amplified size in the studied *Secale* taxa was 653 bp (Table 1) and corresponded to the size of other species.

In contrast, the length of the *trnD* [tRNA-Asp (GUC)]—*trnT* [tRNA-Thr (GGU)] region in the analyzed rye species and subspecies was 963 bp (Table 1), similarly as in *S*. *cereale*– 948 bp [40]. It was slightly shorter than described in wheat (1047 bp) and maize (1185 bp) or couch grass (1200 bp), and also significantly shorter than the corresponding region in barley (1978 bp) [43]. This region, next to *trnT*(UGU)-*trnL*(UAA) 5' exon, was characterized by the highest variability in rye species among all analyzed cpDNA regions and could be successfully used in studies of closely related species.

### Evaluation of sequence variability of mtDNA non-coding regions in rye species

The mitochondrial genomes of plants are characterized by the presence of a relatively large number of group II introns compared to mtDNA of fungi and bacteria [89,90]. Group II introns described so far in plants have been located in the genes encoding proteins [91]. There was one exception noted–the presence of group II intron in the *trnA* gene in several species of the genus *Citrus* and in the wheat genome [92,93]. However, the *trnA* gene is of chloroplast origin and also has an intron in cpDNA [94]. Approximately 1/3 of group II introns is located in the plastid tRNA.

Several plant genera, including *Peperomia* and *Marchantia* also contains group I introns, located in the *coxI* gene [94]. However, there is no correlation between phylogenesis and the presence of this intron, which indicates that it was introduced by horizontal gene transfer, and a fungal species was probably the donor.

In this study, a total of 45 indels have been identified (Table 2) and this number is comparable to the results of other authors in mtDNA of plants. For example, Christensen et al. identified 35 indels in *A*. *thaliana* mtDNA [95], and Ossowski et al. [96] reported that the *A*. *thaliana* mitochondrial genome was characterized by a much higher content of indels in comparison to the nuclear genome.

Unfortunately, there is no sequence data on rye mtDNA, however, the mtDNA sequence in winter wheat (*Triticum aestivum* cv. Chinese Yumai) is almost identical to the spring wheat sequence (*T*. *aestivum* cv. Chinese Spring) [97]. Only 10 indels were identified between two independently acquired sequences, and all variants were found in non-coding regions. A 4-bp indel served as a convenient marker for discriminating the cultivar Chinese Yumai from Chinese Spring [97].

On average, 3 haplotypes were identified among the six analyzed mtDNA sequences (Table 2). Most haplotypes (12) were detected in the *nad4/1-2* region. The most frequently occurring was haplotype 1 in the non-coding *rps12-nad3*(2) region (S9 Table).

Mitochondrial primers amplifying the intergenic *nad1B*-*nad1C* region are located in exon b and c [43]. This region has a length of about 1483 bp in the analyzed rye species and subspecies (Table 2). Its size is comparable with the corresponding region in the couch grass (1600 bp) [60], and pedunculate oak (1550 bp) [43] and is shorter than that described in the Arabidopsis [41]. *Marchantia polymorpha* is one of the species that do not have an intron in the *nad1* gene [94]. The results obtained in this work confirmed the highly conserved nature of this intron group. The *nad1* intron region may nevertheless serve as a useful molecular marker in population studies, because it contains size variants that reveal the population structure in the entire range, e.g., in *Pinus ponderosa* and *P*. *flexilis* [98–100]. Unfortunately, no such differences were found in rye, and the obtained sequences were conserved (Table 2).

The intron located within subunit 4 of the *nad4* gene is considered to be a slowly evolving mitochondrial marker, whose evolution occurs 23 times slower than that of the ITS rDNA sequence [101] Therefore, it is considered more useful in solving the “deeper” phylogenetic relationships. It was successfully used in the phylogenetic analyses of the Brassicaceae family [102]. It was found in this work that this sequence could describe phylogenetic relationships within almost the whole family. Similarly, based on the presented studies, it was confirmed that sequences of this region proved to be the most informative among all tested mtDNA sequences. Twelve haplotypes were described among 35 analyzed taxa, and the haplotypes diversity was the highest (0.867) (Table 2). The length of this intron in *Secale* species is 1642 bp and is significantly shorter than that described in *Arabidopsis* (2103 bp) [41]. The reason why the *nad4/1-2* intron is shorter is not known due to the lack of information on these sequences in rye in the NCBI Genebank. The sequences of this intron, in addition to the *rps12-nad3*(2) intergenic sequence, showed variation at both intraspecific and interspecific levels (Table 2).

Another analyzed intergenic region, *nad4L-orf25*, in rye (626 bp) is of size corresponding to those sequences in *Arabidopsis* (671 bp) and slightly shorter than in wheat (899 bp) [99]. These sequences were conserved in the taxa analyzed (Table 2). This result was confirmed by the data on sugar beet [103], indicating conserved nature of this region in angiosperms. The gene encoding the 4L subunit of NADH dehydrogenase (*nad4L*) in the mitochondrial genome of *Arabidopsis thaliana* is located between exon c of the *nad5* open reading frame and *orf25* [104], in wheat between *nad7* and *rps19* [99].

Two different combinations of primers were used to amplify the intergenic sequences of the *rps12-nad3* region: the first amplified only the intergenic region, the second–the intergenic region and the *nad3* gene. The results obtained in this study indicated the variability of the analyzed intergenic sequences, while the first region proved conserved in *Secale* species and subspecies (Table 2). The length of the *rps12-nad3* intron in the analyzed taxa was 134 bp, and it was much shorter than the intron of *Arabidopsis* (700 bp). In contrast, its size in wheat is 45 bp [99]. The second primer pair amplified the 120-bp region, comparable to the corresponding region in *Arabidopsis* (139 bp).

The *rrn5-rrn18* ribosomal region in the analyzed forms was 166 bp long, slightly shorter than that described in *Arabidopsis* (273 bp) and comparable to the corresponding wheat region (115 bp). This region was also conserved among the analyzed *Secale* species and sub-species (Table 2).

### Evaluation of genetic diversity in the genus *Secale*

Trees obtained using different methods (BI, ML) for all analyzed sequences of non-coding chloroplast and mitochondrial DNA and a collective tree, present a similar topology. There are no visible systematic or geographical relationships on the trees (Figs 1–3). Clades separated on the trees combine various rye subspecies from different locations. The results obtained in this work confirmed the reports of other authors about the lack of monophyletism of *Secale* sp. subspecies resulting from similar values of inter- and intraspecific distance [105]. In contrast to the results presented by Santos [26], our analyses showed that sequences from the same geographical regions did not group in any of the phylogenetic trees.

The existence of 5 clades encompassing various taxa may indicate the introgression between them (Fig 3), as previously described [6,106,107]. Only S. *sylvestre* Hungary and Bulgaria, formed a separate group, which confirmed the latest results on the basis of the SSR and nuclear EST analysis [24] and the results based on the DArTseq analysis [27]. Many authors [3,4,12,108] consider *S*. *sylvestre* as the oldest species, from which all other species evolved. Similarly, in our research, it was the most diverse species of all *Secale* taxa (Fig 3). Hammer [109] argued that *S*. *sylvestre* was developing separately, and its evolution could have begun very early. Unfortunately, the remaining analyzed *S*. *sylvestre* taxa from Poland and Russia grouped in other clades (Clade 1) with *S*. *cereale* and *S*. *strictum* species and subspecies (Fig 3). Similar results were obtained by Ren et al. [110] based on the analysis of microsatellite sequences. They showed similarity of *S*. *sylvestre* to *S*. *strictum* ssp. *africanum* and *anatolicum*. Skuza et al. [18] in turn classified *S*. *sylvestre* with *S*. *cereale* ssp. *segetale* based on mtDNA analysis.

*S*. *vavilovii* is an annual species, similar to *S*. *cereale*, *S*. *sylvestre* and *S*. *segetale*, however, according to Hammer [109], it has evolved with multiannual species: *S*. *africanum*, *S*. *strictum* and *S*. *kuprijanovii*. Research conducted by Jones and Flavell [28] hypothesized the common origin of *S*. *vavilovii*, *africanum* and *cereale* from the *S*. *strictum* line. The results obtained in the current work did not confirm this theory. *S*. *vavilovii* was dispersed and was in the similarity group with both *cereale* and *strictum* (Fig 3). The result of the analysis was partly consistent with the classification of Frederiksen and Petersen [111], who identified only three species within the genus *Secale*: *S*. *sylvestre*, *S*. *strictum* and *S*. *cereale* and included *S*. *vavilovii* to *S*. *cereale*. Similarly, Kobyljanski [112] classified *S*. *vavilovii* as a subspecies of *S*. *cereale*. Bolibok-Bągoszewska et al. [113] also postulated to classify these species together. The placement of *S*. *vavilovii* and *S*. *cereale* in one section is supported by the fact that seven bivalens are observed in the crosses of these two species, which indicates a complete structural similarity between these species [106]. Our result was also confirmed by the earlier data of Al-Beyroutiova et al. [27], who argued that *S*. *vavilovii* could not be considered as a separate species, but only as a subspecies of S. *cereale*. Maraci et al. [24] reached similar conclusions, showing a greater similarity between *S*. *vavilovii* and *S*. *c*. ssp. *cereale* and *segetale* than within the *cereale* species.

The previous results of other authors indicated that the separate and phylogenetic position of *S*. *strictum* subspecies was unquestionable [12]. However, the assumption regarding the origin of *strictum* species is inconsistent with our results, as well as with the results of tandem sequence repeats and AFLP and SSR data [12,13,15]. The *strictum* species group is heterogeneous and shows similarity to *S*. *cereale* subspecies, similarly as in the work of Ren [113]. This is consistent with the hypothesis that cultivated rye evolved from *S*. *strictum* [113–117]. Genetic diversity in the evolutionary process was lower in the *strictum* group than between perennial and annual forms and species. In addition, it has been shown that the perennial forms are morphologically similar and cross easily to form hybrids [3,118].

The taxonomic position of *S*. *strictum* ssp. *ciliatoglume* is also often undermined. Bolibok-Bągoszewska et al. [113] argued to classify it together with *S*. *vavilovii* in the *S*. *cereale* species group. However, the results obtained in the present work did not confirm this theory, because *S*. *strictum* ssp. *ciliatoglume* showed a clear similarity to *S*. *strictum* ssp. *anatolicum* (Figs 1 and 3). Broda et al. [118] also classified *S*. *s*. ssp. *cicliatoglume* in a separate group to *S*. *vavilovii*. Al-Beyroutiova et al. [27] postulated to analyze this taxon more accurately due to the fact that *S*. *s*. ssp. *cicliatoglume* was not associated with other *strictum* subspecies.

Hammer [109] grouped *S*. *strictum* species into one group with *S*. *vavilovii* and *S*. *kuprijanovii*, which was not confirmed in the present study. On the other hand, a large similarity of *S*. *vavilovii* (Poland) to *S*. *s*. ssp. *africanum* (RSA1) could be observed (Figs 2 and 3). The results obtained in the current study confirmed the similarity of *S*. *s*. *strictum* to *S*. *c*. ssp. *kuprijanovii* (Figs 2 and 3), consistent with the adopted classification. Noteworthy is the heterogeneity of the sequence of two analyzed samples of *S*. *strictum* ssp. *africanum*. The results clearly suggested that the origin of these seeds was not homogeneous–the second sample (RSA1) formed a separate subgroup.

*S*. *africanum* species has been most often classified to *S*. *strictum*. Hammer [109] considered *S*. *africanum* and *S*. *strictum* species to be very similar to each other, which was confirmed in this work (Fig 3). However, the latest data based on SNP analyses confirmed our results, classifying *S*. *strictum* and *africanum* as separate species [25]. *S*. *africanum* occurs only in southern Africa, but it can cross with other *Secale* species [13]. The high genetic similarity between *S*. *africanum* and *S*. *strictum* species strengthens the hypothesis that the location of *S*. *africanum* away from other *Secale* taxa should be explained by human activities, and not as a remnant of the originally much larger *Secale* distribution area [111].

S. *cereale* species also did not form groups consistent with the current classification. The dispersion of *cereale* species was also described by Hagenblad et al. [25], who found that genetic grouping of *cereale* species was more dependent on geographical origin than the taxonomic classification. Similarly, Al-Beyroutiova et al. [27] reported insufficient divergence of *cereale* subspecies.

## Conclusions

The use of non-coding sequences of chloroplast and mitochondrial DNA provided new data on genetic diversity within the genus *Secale*. Such sequences have so far not been used in any analysis of phylogenetic relationships in rye. The results obtained in this study clearly indicated disproportions in the available information regarding various non-coding cpDNA regions used in phylogenetic studies, and some of them–due to high variability–can be successfully used in the analyses of closely related species.

The analysis of genetic diversity and phylogenetic relationships of the genus *Secale* shows the lack of monophyletism of *Secale* sp. subspecies resulting from similar inter- and intraspecific distance, which indicates the continuation of gene flow between species. The reason for this may be the relatively recent evolution of rye species that did not allow the full formation of isolation mechanisms.

The lack of a clear genetic diversity between *S*. *cereale* and *S*. *vavilovii* confirmed the assumption that they might share a common ancestor. Furthermore, it allows to conclude—as mentioned by many authors—that *S*. *vavilovii* should be classified as a subspecies of *S*. *cereale*. Moreover, the results obtained in this work confirmed the divergence of *S*. *sylvestre* from other species and subspecies of rye.

The results also indicate regions that may be useful molecular markers in studies on closely related species of the genus *Secale*. These include the non-coding regions of chloroplast DNA: *trnT* (UGU)-*trnL*(UAA)5'exon and *trnD*[tRNA-Asp(GUC)]-*trnT*[tRNA-Thr(GGU)] and non-coding regions of mitochondrial DNA: *nad4/1-2* and *rps12-nad3*(2). However, the chloroplast genome is more useful for this kind of analysis. We presume that the analysis of the complete chloroplast genome sequences in species of rye will be useful and cost-effective for evolutionary and phylogenetic studies.

## Supporting information

S1 Table The list of plant species, origin, accession numbers, NCBI Genebank accession numbers, type and life cycle. (DOCX)

S2 Table Primer list of sequences (5′-3′) for amplification different regions of cpDNA and their references used in this study. (DOCX)

S3 Table Primer list of sequences (5′-3′) for amplification different regions of mtDNA and their references used in this study. (DOCX)

S4 Table PCR reagents for amplification different regions the cpDNA. (DOCX)

S5 Table PCR reagents for amplification different regions the mtDNA. (DOCX)

S6 Table Thermocycling conditions for a PCR amplification of cpDNA noncoding (intron) regions. (DOCX)

S7 Table Thermocycling conditions for a PCR amplification of mtDNA noncoding (intron) regions. (DOCX)

S8 Table The type and number of haplotypes obtained for the noncoding cpDNA sequences. (DOCX)

S9 Table The type and number of haplotypes obtained for the noncoding mtDNA sequences. (DOCX)
