# Supplementary material for: Genetic diversity and relationship between cultivated, weedy and wild rye species as revealed by chloroplast and mitochondrial DNA non-coding regions analysis
Source: PLoS One. 2019 Feb 27;14(2):e0213023. doi: 10.1371/journal.pone.0213023 (PMC6392296; doi:10.1371/journal.pone.0213023)
Supplement: S1 Table — (DOCX) [file pone.0213023.s001.docx]

| No. | Species | Origin | NCBI Genbank Accession numbers | | | | Accession number | Type^a^ | Life Cycle^a^ |
| --- | --- | --- | --- | --- | --- | --- | --- | --- | --- |
|  |  |  | ^b^cpDNA-regions | | ^c^mtDNA-regions | |  |  |  |
| 1 | *Secale cereale* ssp. *afghanicum* | Armenia | 1 | MH893827 | 5 | MH893967 | PI 618662 | We | A |
|  |  |  | 2 | MH893862 | 6 | MH894002 |  |  |  |
|  |  |  | 3 | MH893897 | 7 | MH894037 |  |  |  |
|  |  |  | 4 | MH893932 | 8 | MH894072 |  |  |  |
|  |  |  |  |  | 9 | MH894107 |  |  |  |
|  |  |  |  |  | 10 | MH894142 |  |  |  |
| 2 | *Secale cereale* ssp. *ancestrale* | Japan | 1 | MH893828 | 5 | MH893968 | CI SE 107 | We | A |
|  |  |  | 2 | MH893863 | 6 | MH894003 |  |  |  |
|  |  |  | 3 | MH893898 | 7 | MH894038 |  |  |  |
|  |  |  | 4 | MH893933 | 8 | MH894073 |  |  |  |
|  |  |  |  |  | 9 | MH894108 |  |  |  |
|  |  |  |  |  | 10 | MH894143 |  |  |  |
| 3 |  | Russia | 1 | MH893829 | 5 | MH893969 | PI 445975 |  |  |
|  |  |  | 2 | MH893864 | 6 | MH894004 |  |  |  |
|  |  |  | 3 | MH893899 | 7 | MH894039 |  |  |  |
|  |  |  | 4 | MH893934 | 8 | MH894074 |  |  |  |
|  |  |  |  |  | 9 | MH894109 |  |  |  |
|  |  |  |  |  | 10 | MH894144 |  |  |  |
| 4 |  | Turkey | 1 | MH893830 | 5 | MH893970 | PI 618666 |  |  |
|  |  |  | 2 | MH893865 | 6 | MH894005 |  |  |  |
|  |  |  | 3 | MH893900 | 7 | MH894040 |  |  |  |
|  |  |  | 4 | MH893935 | 8 | MH894075 |  |  |  |
|  |  |  |  |  | 9 | MH894110 |  |  |  |
|  |  |  |  |  | 10 | MH894145 |  |  |  |
| 5 |  | USA | 1 | MH893831 | 5 | MH893971 | PI 445976 |  |  |
|  |  |  | 2 | MH893866 | 6 | MH894006 |  |  |  |
|  |  |  | 3 | MH893901 | 7 | MH894041 |  |  |  |
|  |  |  | 4 | MH893936 | 8 | MH894076 |  |  |  |
|  |  |  |  |  | 9 | MH894111 |  |  |  |
|  |  |  |  |  | 10 | MH894146 |  |  |  |
| 6 | *Secale cereale* ssp. *cereale* | Canada | 1 | MH893832 | 5 | MH893972 | PI 590948 | C | A |
|  |  |  | 2 | MH893867 | 6 | MH894007 |  |  |  |
|  |  |  | 3 | MH893902 | 7 | MH894042 |  |  |  |
|  |  |  | 4 | MH893937 | 8 | MH894077 |  |  |  |
|  |  |  |  |  | 9 | MH894112 |  |  |  |
|  |  |  |  |  | 10 | MH894147 |  |  |  |
| 7 |  | Pakistan | 1 | MH893833 | 5 | MH893973 | PI 561809 |  |  |
|  |  |  | 2 | MH893868 | 6 | MH894008 |  |  |  |
|  |  |  | 3 | MH893903 | 7 | MH894043 |  |  |  |
|  |  |  | 4 | MH893938 | 8 | MH894078 |  |  |  |
|  |  |  |  |  | 9 | MH894113 |  |  |  |
|  |  |  |  |  | 10 | MH894148 |  |  |  |
| 8 |  | USA | 1 | MH893834 | 5 | MH893974 | PI 628642 |  |  |
|  |  |  | 2 | MH893869 | 6 | MH894009 |  |  |  |
|  |  |  | 3 | MH893904 | 7 | MH894044 |  |  |  |
|  |  |  | 4 | MH893939 | 8 | MH894079 |  |  |  |
|  |  |  |  |  | 9 | MH894114 |  |  |  |
|  |  |  |  |  | 10 | MH894149 |  |  |  |
| 9 |  | Tajikistan | 1 | MH893835 | 5 | MH893975 | PI 639383 |  |  |
|  |  |  | 2 | MH893870 | 6 | MH894010 |  |  |  |
|  |  |  | 3 | MH893905 | 7 | MH894045 |  |  |  |
|  |  |  | 4 | MH893940 | 8 | MH894080 |  |  |  |
|  |  |  |  |  | 9 | MH894115 |  |  |  |
|  |  |  |  |  |  | MH894150 |  |  |  |
| 10 | *Secale cereale* ssp. *segetale* | Azerbaijan | 1 | MH893836 | 5 | MH893976 | PI 267102 | We | A |
|  |  |  | 2 | MH893871 | 6 | MH894011 |  |  |  |
|  |  |  | 3 | MH893906 | 7 | MH894046 |  |  |  |
|  |  |  | 4 | MH893941 | 8 | MH894081 |  |  |  |
|  |  |  |  |  | 9 | MH894116 |  |  |  |
|  |  |  |  |  | 10 | MH894151 |  |  |  |
| 11 |  | Russia | 1 | MH893837 | 5 | MH893977 | PI 283982 |  |  |
|  |  |  | 2 | MH893871 | 6 | MH894012 |  |  |  |
|  |  |  | 3 | MH893907 | 7 | MH894047 |  |  |  |
|  |  |  | 4 | MH893942 | 8 | MH894082 |  |  |  |
|  |  |  |  |  | 9 | MH894117 |  |  |  |
|  |  |  |  |  | 10 | MH894152 |  |  |  |
| 12 |  | Kazakhstan | 1 | MH893838 | 5 | MH893978 | PI 326286 |  |  |
|  |  |  | 2 | MH893872 | 6 | MH894013 |  |  |  |
|  |  |  | 3 | MH893908 | 7 | MH894048 |  |  |  |
|  |  |  | 4 | MH893943 | 8 | MH894083 |  |  |  |
|  |  |  |  |  | 9 | MH894118 |  |  |  |
|  |  |  |  |  | 10 | MH8941453 |  |  |  |
| 13 |  | Turkey | 1 | MH893839 | 5 | MH893979 | PI 618673 |  |  |
|  |  |  | 2 | MH893873 | 6 | MH894014 |  |  |  |
|  |  |  | 3 | MH893909 | 7 | MH894049 |  |  |  |
|  |  |  | 4 | MH893944 | 8 | MH894084 |  |  |  |
|  |  |  |  |  | 9 | MH894119 |  |  |  |
|  |  |  |  |  |  | MH894154 |  |  |  |
| 14 | *Secale cereale* ssp. *dighoricum* | Sweden | 1 | MH893840 | 5 | MH893980 | PI 618667 | We | A |
|  |  |  | 2 | MH893874 | 6 | MH894015 |  |  |  |
|  |  |  | 3 | MH893910 | 7 | MH894050 |  |  |  |
|  |  |  | 4 | MH893945 | 8 | MH894085 |  |  |  |
|  |  |  |  |  | 9 | MH894120 |  |  |  |
|  |  |  |  |  | 10 | MH8941455 |  |  |  |
| 15 |  | Russia | 1 | MH893841 | 5 | MH893981 | PI 618668 |  |  |
|  |  |  | 2 | MH893875 | 6 | MH894016 |  |  |  |
|  |  |  | 3 | MH893911 | 7 | MH894051 |  |  |  |
|  |  |  | 4 | MH893946 | 8 | MH894086 |  |  |  |
|  |  |  |  |  | 9 | MH894121 |  |  |  |
|  |  |  |  |  | 10 | MH8941456 |  |  |  |
| 16 | *Secale cereale* ssp. *rigidum* | Turkey | 1 | MH893842 | 5 | MH893982 | PI 618669 | We | A |
|  |  |  | 2 | MH893876 | 6 | MH894017 |  |  |  |
|  |  |  | 3 | MH893912 | 7 | MH894052 |  |  |  |
|  |  |  | 4 | MH893947 | 8 | MH894087 |  |  |  |
|  |  |  |  |  | 9 | MH894122 |  |  |  |
|  |  |  |  |  | 10 | MH894157 |  |  |  |
| 17 |  | Poland | 1 | MH893843 | 5 | MH893983 | 124197 |  |  |
|  |  |  | 2 | MH893877 | 6 | MH894018 |  |  |  |
|  |  |  | 3 | MH893913 | 7 | MH894053 |  |  |  |
|  |  |  | 4 | MH893948 | 8 | MH894088 |  |  |  |
|  |  |  |  |  | 9 | MH894123 |  |  |  |
|  |  |  |  |  | 10 | MH8941458 |  |  |  |
| 18 | *Secale strictum* ssp. *africanum* | RSA | 1 | MH893844 | 5 | MH893984 | PI 630963 | Wi | P |
|  |  |  | 2 | MH893878 | 6 | MH894019 |  |  |  |
|  |  |  | 3 | MH893914 | 7 | MH894054 |  |  |  |
|  |  |  | 4 | MH893949 | 8 | MH894089 |  |  |  |
|  |  |  |  |  | 9 | MH894124 |  |  |  |
|  |  |  |  |  | 10 | MH8941459 |  |  |  |
| 19 |  | RSA | 1 | MH893845 | 5 | MH893985 | PI 630964 |  |  |
|  |  |  | 2 | MH893879 | 6 | MH894020 |  |  |  |
|  |  |  | 3 | MH893915 | 7 | MH894056 |  |  |  |
|  |  |  | 4 | MH893950 | 8 | MH894090 |  |  |  |
|  |  |  |  |  | 9 | MH894125 |  |  |  |
|  |  |  |  |  | 10 | MH894160 |  |  |  |
| 20 | *Secale strictum* ssp. *anatolicum* | USA | 1 | MH893846 | 5 | MH893986 | PI 445973 | Wi | P |
|  |  |  | 2 | MH893880 | 6 | MH894021 |  |  |  |
|  |  |  | 3 | MH893916 | 7 | MH894057 |  |  |  |
|  |  |  | 4 | MH893951 | 8 | MH894091 |  |  |  |
|  |  |  |  |  | 9 | MH894126 |  |  |  |
|  |  |  |  |  | 10 | MH894161 |  |  |  |
| 21 |  | Canada | 1 | MH893847 | 5 | MH893987 | PI 445974 |  |  |
|  |  |  | 2 | MH893881 | 6 | MH894022 |  |  |  |
|  |  |  | 3 | MH893917 | 7 | MH894058 |  |  |  |
|  |  |  | 4 | MH893952 | 8 | MH894092 |  |  |  |
|  |  |  |  |  | 9 | MH894127 |  |  |  |
|  |  |  |  |  | 10 | MH8941462 |  |  |  |
| 22 |  | Turkey | 1 | MH893848 | 5 | MH893988 | PI 630965 |  |  |
|  |  |  | 2 | MH893882 | 6 | MH894023 |  |  |  |
|  |  |  | 3 | MH893918 | 7 | MH894059 |  |  |  |
|  |  |  | 4 | MH893953 | 8 | MH894093 |  |  |  |
|  |  |  |  |  | 9 | MH894128 |  |  |  |
|  |  |  |  |  | 10 | MH894163 |  |  |  |
| 23 | *Secale strictum* ssp. *kuprijanovii* | Poland | 1 | MH893849 | 5 | MH893989 | PI 31328 | Wi | P |
|  |  |  | 2 | MH893883 | 6 | MH894024 |  |  |  |
|  |  |  | 3 | MH893919 | 7 | MH894060 |  |  |  |
|  |  |  | 4 | MH893954 | 8 | MH894094 |  |  |  |
|  |  |  |  |  | 9 | MH894129 |  |  |  |
|  |  |  |  |  | 10 | MH8941464 |  |  |  |
| 24 | *Secale strictum* ssp *strictum* | Poland | 1 | MH893850 | 5 | MH893990 | PI 630971 | Wi | P |
|  |  |  | 2 | MH893884 | 6 | MH894025 |  |  |  |
|  |  |  | 3 | MH893920 | 7 | MH894061 |  |  |  |
|  |  |  | 4 | MH893955 | 8 | MH894095 |  |  |  |
|  |  |  |  |  | 9 | MH894130 |  |  |  |
|  |  |  |  |  | 10 | MH894165 |  |  |  |
| 25 |  | Hungary | 1 | MH893851 | 5 | MH893991 | PI 272338 |  |  |
|  |  |  | 2 | MH893885 | 6 | MH894026 |  |  |  |
|  |  |  | 3 | MH893921 | 7 | MH894062 |  |  |  |
|  |  |  | 4 | MH893956 | 8 | MH894096 |  |  |  |
|  |  |  |  |  | 9 | MH894131 |  |  |  |
|  |  |  |  |  | 10 | MH894166 |  |  |  |
| 26 |  | Iraq | 1 | MH893852 | 5 | MH893992 | PI 253956 |  |  |
|  |  |  | 2 | MH893886 | 6 | MH894027 |  |  |  |
|  |  |  | 3 | MH893922 | 7 | MH894063 |  |  |  |
|  |  |  | 4 | MH893957 | 8 | MH894097 |  |  |  |
|  |  |  |  |  | 9 | MH894132 |  |  |  |
|  |  |  |  |  | 10 | MH894167 |  |  |  |
| 27 | *Secale strictum* ssp. *ciliatoglume* | Poland | 1 | MH893853 | 5 | MH893993 | PI 630967 | Wi | P |
|  |  |  | 2 | MH893887 | 6 | MH894028 |  |  |  |
|  |  |  | 3 | MH893923 | 7 | MH894064 |  |  |  |
|  |  |  | 4 | MH893958 | 8 | MH894098 |  |  |  |
|  |  |  |  |  | 9 | MH894133 |  |  |  |
|  |  |  |  |  | 10 | MH894168 |  |  |  |
| 28 | *Secale vavilovii* | Afghanistan | 1 | MH893854 | 5 | MH893994 | PI 57364 | Wi | A |
|  |  |  | 2 | MH893888 | 6 | MH894029 |  |  |  |
|  |  |  | 3 | MH893924 | 7 | MH894065 |  |  |  |
|  |  |  | 4 | MH893959 | 8 | MH894099 |  |  |  |
|  |  |  |  |  | 9 | MH894134 |  |  |  |
|  |  |  |  |  | 10 | MH894169 |  |  |  |
| 29 |  | Russia | 1 | MH893855 | 5 | MH893995 | PI 573648 |  |  |
|  |  |  | 2 | MH893889 | 6 | MH894030 |  |  |  |
|  |  |  | 3 | MH893925 | 7 | MH894064 |  |  |  |
|  |  |  | 4 | MH893960 | 8 | MH894100 |  |  |  |
|  |  |  |  |  | 9 | MH894135 |  |  |  |
|  |  |  |  |  | 10 | MH894170 |  |  |  |
| 30 |  | Hungary | 1 | MH893856 | 5 | MH893996 | PI 28842 |  |  |
|  |  |  | 2 | MH893890 | 6 | MH894031 |  |  |  |
|  |  |  | 3 | MH893926 | 7 | MH894065 |  |  |  |
|  |  |  | 4 | MH893961 | 8 | MH894101 |  |  |  |
|  |  |  |  |  | 9 | MH894136 |  |  |  |
|  |  |  |  |  | 10 | MH894171 |  |  |  |
| 31 |  | Poland | 1 | MH893857 | 5 | MH893997 | 831/96 |  |  |
|  |  |  | 2 | MH893891 | 6 | MH894032 |  |  |  |
|  |  |  | 3 | MH893927 | 7 | MH894066 |  |  |  |
|  |  |  | 4 | MH893962 | 8 | MH894102 |  |  |  |
|  |  |  |  |  | 9 | MH894137 |  |  |  |
|  |  |  |  |  | 10 | MH894172 |  |  |  |
| 32 | *Secale sylvestre* | Russia | 1 | MH893858 | 5 | MH893998 | 6047 | Wi | P |
|  |  |  | 2 | MH893892 | 6 | MH894033 |  |  |  |
|  |  |  | 3 | MH893928 | 7 | MH894067 |  |  |  |
|  |  |  | 4 | MH893963 | 8 | MH894103 |  |  |  |
|  |  |  |  |  | 9 | MH894138 |  |  |  |
|  |  |  |  |  | 10 | MH894173 |  |  |  |
| 33 |  | Hungary | 1 | MH893859 | 5 | MH893999 | PI 615332 |  |  |
|  |  |  | 2 | MH893893 | 6 | MH894034 |  |  |  |
|  |  |  | 3 | MH893929 | 7 | MH894068 |  |  |  |
|  |  |  | 4 | MH893964 | 8 | MH894104 |  |  |  |
|  |  |  |  |  | 9 | MH894139 |  |  |  |
|  |  |  |  |  | 10 | MH894174 |  |  |  |
| 34 |  | Bulgaria | 1 | MH893860 | 5 | MH894000 | PI 618675 |  |  |
|  |  |  | 2 | MH893894 | 6 | MH894035 |  |  |  |
|  |  |  | 3 | MH893930 | 7 | MH894069 |  |  |  |
|  |  |  | 4 | MH893965 | 8 | MH894105 |  |  |  |
|  |  |  |  |  | 9 | MH894140 |  |  |  |
|  |  |  |  |  | 10 | MH894175 |  |  |  |
| 35 |  | Poland | 1 | MH893861 | 5 | MH894001 | PI 618676 |  |  |
|  |  |  | 2 | MH893895 | 6 | MH894035 |  |  |  |
|  |  |  | 3 | MH893931 | 7 | MH894070 |  |  |  |
|  |  |  | 4 | MH893966 | 8 | MH894105 |  |  |  |
|  |  |  |  |  | 9 | MH894140 |  |  |  |
|  |  |  |  |  | 10 | MH894176 |  |  |  |

^a^ Wi- wild, We – weedy, C - cultivated, P – parennial, A – annual

^b^cp-DNA regions:1- *atpB-rbcL intergenic spacer*, 2- *trnT(UGU)-trnL5'exon intergenic spacer,* 3- *trnL(UAA) intron intergenic spacer*, 4- *trnD[tRNA-Asp(GUC)]-trnT [tRNA-Thr(GGU)]*;

^c^mt-DNA regions: 5- *nad1 exon B-nad1 exon C intron intergenic spacer*, 6-*nad4/1-2 intergenic spacer*,7-*nad4L-orf25 intergenic spacer*,8-*rps12-1/nad3-2 intergenic spacer*,9- *rps12-2/nad3-1 intergenic spacer*,10-*rrn5/rrn18-1 intergenic spacer*
