# Supplementary material for: Genetic diversity and relationship between cultivated, weedy and wild rye species as revealed by chloroplast and mitochondrial DNA non-coding regions analysis
Source: PLoS One. 2019 Feb 27;14(2):e0213023. doi: 10.1371/journal.pone.0213023 (PMC6392296; doi:10.1371/journal.pone.0213023)
Supplement: S2 Table — (DOCX) [file pone.0213023.s002.docx]

| **Type of DNA** | **Locus** | **Direction** | **Sequence 5’- 3’** | **Reference** |
| --- | --- | --- | --- | --- |
| cpDNA | *atpB-rbcL* | Forward | ACATCKARTACKGGACCAATAA | (Chiang et al., 1998) |
|  |  | Reverse | AACACCAGCTTTRAATCCAA | (Chiang et al., 1998) |
|  | *trnT* (UGU) -*trnL* (UAA) exon | Forward | CATTACAAATGCGATGCTCT | (Taberlet et al., 1991) |
|  |  | Reverse | TCTACCGATTTCGCCATATC | (Taberlet et al., 1991) |
|  | *trnL* (UAA) intron | Forward | CGAAATCGGTAGACGCTACG | (Taberlet et al., 1991) |
|  |  | Reverse | GGGGATAGAGGGACTTGAAC | (Taberlet et al., 1991) |
|  | *trnL* (UAA) 3' exon-*trnF* (GAA) | Forward | GGTTCAAGTCCCTCTATCCC | (Taberlet et al., 1991) |
|  |  | Reverse | ATTTGAACTGGTGACACGAG | (Taberlet et al., 1991) |
|  | *trnD*[tRNA–Asp(GUC)]-*trnT*[tRNA–Thr(GGU)] | Forward | ACCAATTGAACTACAATCCC | (Demesure et al., 1995) |
|  |  | Reverse | CCCTTTTAACTCAGTGGTAG | (Demesure et al., 1995) |
