# Supplementary material for: Genetic diversity and relationship between cultivated, weedy and wild rye species as revealed by chloroplast and mitochondrial DNA non-coding regions analysis
Source: PLoS One. 2019 Feb 27;14(2):e0213023. doi: 10.1371/journal.pone.0213023 (PMC6392296; doi:10.1371/journal.pone.0213023)
Supplement: S3 Table — (DOCX) [file pone.0213023.s003.docx]

| **Type of DNA** | **Locus** | **Direction** | **Sequence 5’- 3’** | **Reference** |
| --- | --- | --- | --- | --- |
| mtDNA | *nad1* exon B-*nad1* exon C intron | Forward | GCATTACGATCTGCAGCTCA | (Demesure et al., 1995) |
|  |  | Reverse | GGAGCTCGATTAGTTTCTGC | (Demesure et al., 1995) |
|  | *nad4*/1-2 | Forward | CAGTGGGTTGGTCTGGTAATG | (Demesure et al., 1995) |
|  |  | Reverse | TCATATGGGCTACTGAGGAG | (Demesure et al., 1995) |
|  | *nad4L-orf25* | Forward | CTGTYTTTTCGCACTTAGGC | (Duminil et al., 2002) |
|  |  | Reverse | GTCCGRGGTACTATTGCTGT | (Duminil et al., 2002) |
|  | *rps12-1/nad3-2* | Forward | TTTCTTCTCTACCATGACGA | (Duminil et al., 2002) |
|  |  | Reverse | TGATCCYACTCGGTSTTCCT | (Duminil et al., 2002) |
|  | *rps12-2/nad3-1* | Forward | ACCATATTTDGATCTGCCDC | (Duminil et al., 2002) |
|  |  | Reverse | YACGATHGGATTTCTMTATG | (Duminil et al., 2002) |
|  | *rrn5/rrn18-1* | Forward | GAGGTCGGAATGGGATCGGG | (Duminil et al., 2002) |
|  |  | Reverse | GGGTGAAGTCGTAACAAGGT | (Duminil et al., 2002) |
