# Supplementary material for: Genetic diversity and relationship between cultivated, weedy and wild rye species as revealed by chloroplast and mitochondrial DNA non-coding regions analysis
Source: PLoS One. 2019 Feb 27;14(2):e0213023. doi: 10.1371/journal.pone.0213023 (PMC6392296; doi:10.1371/journal.pone.0213023)
Supplement: S4 Table — (DOCX) [file pone.0213023.s004.docx]

| Regions of cpDNA | Reagents quantities and final concentration |
| --- | --- |
| *atpB* - *rbcL* | 100 ng DNA, 1xPCR buffer, 0.1 mM dNTPmix, 2.0 mM MgCl_2_, 0.2 mM each primer, 1 U *Taq* Polymerase, 0.1 mg/mL BSA |
| *trnT*(UGU) - *trnL*(UAA)5' exon | 50 ng DNA, 1xPCR buffer, 0.2 mM dNTPmix, 3.0 mM MgCl_2_, 1.0 mM each primer, 1 U *Taq* Polymerase |
| *trnL* (UAA) intron | 50 ng DNA, 1xPCR buffer, 0.2 mM dNTPmix, 3.0 mM MgCl_2_, 0.2 mM each primer, 1 U *Taq* Polymerase |
| *trnL*(UAA)3' exon- *trnF*(GAA) | 150 ng DNA, 1xPCR buffer, 0.2 mM dNTPmix, 3.0 mM MgCl_2_, 1.0 mM each primer, 1 U *Taq* Polymerase |
| *trnD*[tRNA–Asp(GUC)] - *trnT*[tRNA–Thr(GGU)] | 100 ng DNA, 1xPCR buffer, 0.1 mM dNTPmix, 2.5 mM MgCl_2_, 1.0 mM each primer, 1 U *Taq* Polymerase, 0.1 mg/mL BSA |
