# Supplementary material for: Genetic diversity and relationship between cultivated, weedy and wild rye species as revealed by chloroplast and mitochondrial DNA non-coding regions analysis
Source: PLoS One. 2019 Feb 27;14(2):e0213023. doi: 10.1371/journal.pone.0213023 (PMC6392296; doi:10.1371/journal.pone.0213023)
Supplement: S5 Table — (DOCX) [file pone.0213023.s005.docx]

| Regions of mtDNA | Reagents quantities and final concentration |
| --- | --- |
| *nad1* exon B  *nad1* exon C intron | 250 ng DNA, 1xPCR buffer, 0.2 mM dNTPmix, 2.5 mM MgCl_2_, 0.5 mM each primer, 1 U *Taq* Polymerase, 0.05 mg/mL BSA |
| *nad4*/1-2 | 250 ng DNA, 1xPCR buffer, 0.2 mM dNTPmix, 2.5 mM MgCl_2_, 0.5 mM each primer, 1 U *Taq* Polymerase, 0.05 mg/mL BSA |
| *nad4L-orf25* | 275 ng DNA, 1xPCR buffer, 0.2 mM dNTPmix, 2.5 mM MgCl_2_, 0.55 mM each primer, 1 U *Taq* Polymerase, 0.05 mg/mL BSA |
| *rps12-1/nad3*(2) | 150 ng DNA, 1xPCR buffer, 0.1 mM dNTPmix, 4.0 mM MgCl_2_, 0.3 mM each primer, 1.5 U *Taq* Polymerase |
| *rps12-1/nad3*(1) | 75 ng DNA, 1xPCR buffer, 0.2 mM dNTPmix, 3.0 mM MgCl_2_, 0.5 mM each primer, 1.5 U *Taq* Polymerase |
| *rrn5/rrn18-1* | 100 ng DNA, 1xPCR buffer, 0.2 mM dNTPmix, 3.5 mM MgCl_2_, 0.2 mM each primer, 1 U *Taq* Polymerase, 0.05 mg/mL BSA |
