# Supplementary material for: Genetic diversity and relationship between cultivated, weedy and wild rye species as revealed by chloroplast and mitochondrial DNA non-coding regions analysis
Source: PLoS One. 2019 Feb 27;14(2):e0213023. doi: 10.1371/journal.pone.0213023 (PMC6392296; doi:10.1371/journal.pone.0213023)
Supplement: S6 Table — (DOCX) [file pone.0213023.s006.docx]

| Phase of PCR | *atpB-rbcL* | | *trnT* (UGU)-trnL (UAA) 5’exon | | *trnL* (UAA) intron | | *trnD* [tRNA–Asp(GUC)]-  *trnT* [tRNA–Thr(GGU)] | |
| --- | --- | --- | --- | --- | --- | --- | --- | --- |
|  | Temperature  [^0^C] | Time | Temperature  [^0^C] | Time | Temperature  [^0^C] | Time | Temperature  [^0^C] | Time |
| Initial denaturation | 94 | 4 min | 94 | 3 min | 94 | 3 min | 94 | 4 min |
| Denaturation | 92 | 45 s | 94 | 1 min | 94 | 1 min | 92 | 45 s |
| Primer annealing | 53.5 | 45 s | 55 | 1 min | 62 | 1 min | 53.5 | 45 s |
| Primer extension | 72 | 2 min | 72 | 2 min | 72 | 2 min | 72 | 2 min |
| Final extension | 72 | 10 min | 72 | 10 min | 72 | 10 min | 72 | 10 min |
| Number of cycles | 30 | | 35 | | 35 | | 30 | |
