# Supplementary material for: Genetic diversity and relationship between cultivated, weedy and wild rye species as revealed by chloroplast and mitochondrial DNA non-coding regions analysis
Source: PLoS One. 2019 Feb 27;14(2):e0213023. doi: 10.1371/journal.pone.0213023 (PMC6392296; doi:10.1371/journal.pone.0213023)
Supplement: S7 Table — (DOCX) [file pone.0213023.s007.docx]

| Phase of PCR | *nad1* exon B  *nad1* exon C intron | | *nad4*/1-2 | | *nad4L-orf25* | | *rps12-1/nad3*(2) | | *rps12-1/nad3*(1) | | *rrn5/rrn18-1* | |
| --- | --- | --- | --- | --- | --- | --- | --- | --- | --- | --- | --- | --- |
|  | Temperature  [^0^C] | Time | Temperature  [^0^C] | Time | Temperature  [^0^C] | Time | Temperature  [^0^C] | Time | Temperature  [^0^C] | Time | Temperature  [^0^C] | Time |
| Initial denaturation | 94 | 1min | 94 | 1min | 94 | 1min | 94 | 12 min | 94 | 12 min | 94 | 12 min |
| Denaturation | 94 | 45 s | 94 | 45 s | 94 | 45 s | 94 | 45 s | 94 | 45 s | 94 | 45 s |
| Primer annealing | 52 | 45 s | 52 | 45 s | 51.5 | 45 s | 52 | 45 s | 52 | 45 s | 52 | 45 s |
| Primer extension | 72 | 1 min | 72 | 1 min | 72 | 1 min | 72 | 1 min | 72 | 1min | 72 | 1 min |
| Final extension | 72 | 10 min | 72 | 10 min | 72 | 10 min | 72 | 10 min | 72 | 10 min | 72 | 10 min |
| Number of cycles | 40 | | 40 | | 40 | | 40 | | 40 | | 40 | |
