# Supplementary material for: Genetic diversity and relationship between cultivated, weedy and wild rye species as revealed by chloroplast and mitochondrial DNA non-coding regions analysis
Source: PLoS One. 2019 Feb 27;14(2):e0213023. doi: 10.1371/journal.pone.0213023 (PMC6392296; doi:10.1371/journal.pone.0213023)
Supplement: S8 Table — (DOCX) [file pone.0213023.s008.docx]

| ***atpB-rbcL*** | | | | **Type of Haplotype** | **No. of Haplotype** |
| --- | --- | --- | --- | --- | --- |
| **Species /Subspcecies** | **Country of origin** | | |  |  |
| *S*. *cereale ssp. afghanicum* | Armenia | | | 1 | 27 |
| *S*. *cereale ssp. ancestrale* | Turkey, USA | | |  |  |
| *S*. *cereale ssp. cereale* | Canada, Pakistan, Tajikistan | | |  |  |
| *S*. *cereale ssp. segetale* | Azerbaijan, Russia, Kazakhstan, Turkey | | |  |  |
| *S*. *cereale ssp. dighoricum* | Sweden, Russia | | |  |  |
| *S*. *cereale ssp. rigidum* | Turkey, Poland | | |  |  |
| *S*. *strictum ssp. anatolicum* | USA, Canada, Turkey | | |  |  |
| *S*. *strictum ssp. kuprijanovii* | Poland | | |  |  |
| *S*. *strictum ssp strictum* | Poland, Hungary, Iraq | | |  |  |
| *S*. *strictum ssp. ciliatoglume* | Poland | | |  |  |
| *S*. *vavilovii* | Russia, Bulgaraia, Poland | | |  |  |
| *S*. *sylvestre* | Russia, Bulgaria | | |  |  |
| *S. cereale ssp. ancestrale* | Japan | | | 2 | 1 |
| *S*. *cereale ssp. ancestrale* | Russia | | | 3 | 1 |
| *S*. *cereale ssp. cereale* | USA | | | 4 | 1 |
| *S*. *strictum ssp. africanum* | South Africa-RSA, SouthAfricaRSA1 | | | 5 | 2 |
| *S*. *sylvestre* | Bulgaria | | | 6 | 1 |
| *S*. *vavilovii* | Afghanistan | | | 7 | 1 |
| *S*. *vavilovii* | Hungary | | | 8 | 1 |
| **Total No. of Haplotype** | | | | | **35** |
| ***trnT*(UGU)-*trnL*(UAA)5' exon** | | | | **Type of Haplotype** | **No. of Haplotype** |
| **Species /Subspcecies** | **Country of origin** | | | 1 | 23 |
| *S.cereale ssp. afghanicum* | Armenia | | |  |  |
| *S*. *cereale ssp. ancestrale* | Japan, Russia, Turkey, USA | | |  |  |
| *S*. *cereale ssp. cereale* | Canada, Pakistan, USA, Tajikistan | | |  |  |
| *S*. *cereale spp. segetale* | Russia, Kazakhstan, Turkey | | |  |  |
| *S*. *cereale ssp. dighoricum* | Sweden | | |  |  |
| *S*. *cereale ssp. rigidum* | Turkey | | |  |  |
| *S*. *strictum ssp. africanum* | South Africa- RSA1 | | |  |  |
| *S*. *strictum ssp. anatolicum* | USA, Turkey | | |  |  |
| *S*. *strictum ssp. strictum* | Hungary, Iraq | | |  |  |
| *S*. *vavilovii* | Afghanistan, Poland | | |  |  |
| *S*. *sylvestre* | Russia, Bulgaria | | |  |  |
| *S*. *sylvestre* | Hungary | | | 2 | 1 |
| *S*. *sylvestre* | Poland | | | 3 | 1 |
| *S*. *cereale spp. segetale* | Azerbaijan | | | 4 | 1 |
| *S*. *strictum ssp. strictum* | Poland | | | 5 | 2 |
| *S*. *strictum ssp. kuprijanovii* | Poland | | |  |  |
| *S*. *strictum ssp. anatolicum* | Canada | | | 6 | 1 |
| *S*. *vavilovii* | Hungary | | | 7 | 1 |
| *S*. *strictum ssp. africanum* | South Africa- RSA | | | 8 | 1 |
| *S*. *strictum ssp. ciliatoglume* | Poland | | | 9 | 1 |
| *S*. *cereale ssp. dighoricum* | Russia | | | 10 | 1 |
| *S*. *cereale ssp. rigidum* | Poland | | | 11 | 1 |
| *S*. *vavilovii* | Russia | | | 12 | 1 |
| **Total No. of Haplotype** | | | | | **35** |
| ***trnL*(UAA) intron** | | | | **Type of Haplotype** | **No. of Haplotype** |
| **Species /Subspcecies** | | **Country of origin** | | 1 | 26 |
| *S*. *cereale ssp. afghanicum* | | Armenia | |  |  |
| *S*. *cereale ssp. ancestrale* | | Japan, Russia, Turkey, USA | |  |  |
| *S*. *cereale ssp. cereal* | | Canada, Pakistan, USA | |  |  |
| *S*. *cereale ssp. segetale* | | Azerbaijan, Russia, Kazakhstan | |  |  |
| *S*. *cereale ssp. dighoricum* | | Sweden | |  |  |
| *S*. *cereale ssp. rigidum* | | Turkey | |  |  |
| *S*. *strictum ssp. africanum* | | South Africa- RSA, South Africa- RSA1 | |  |  |
| *S*. *strictum ssp. anatolicum* | | Canada, Turkey | |  |  |
| *S*. *strictum ssp. kuprijanovii* | | Poland | |  |  |
| *S*. *strictum ssp strictum* | | Poland, Hungary, Iraq | |  |  |
| *S*. *strictum ssp. ciliatoglume* | | Poland | |  |  |
| *S*. *vavilovii* | | Afghanistan, Hungary, Poland | |  |  |
| *S*. *sylvestre* | | Russia, Poland | |  |  |
| *S*. *cereale ssp. dighoricum* | | Russia | | 2 | 1 |
| *S*. *sylvestre* | | Bulgaria | | 3 | 1 |
| *S. sylvestre,* | | Hungary | | 4 | 2 |
| *S*. *cereal ssp. rigidum* | | Poland | |  |  |
| *S*. *strictum ssp. anatolicum,* | | Turkey | | 5 | 5 |
| *S*. *cereale spp. segetale* | | Turkey | |  |  |
| *S*. *cereale ssp. cereale* | | Tajikistan | |  |  |
| *S*. *strictum ssp. anatolicum* | | USA | |  |  |
| *S*. *vavilovii* | | Russia | |  |  |
| **Total No. of Haplotype** | | | | | **35** |
| ***trnD*[tRNA–Asp(GUC)]-*trnT*[tRNA–Thr(GGU)]** | | | | **Type of Haplotype** | **No. of Haplotype** |
| **Species /Subspcecies** | | | **Country of origin** |  |  |
| *S*. *cereale ssp. afghanicum* | | | Armenia | 1 | 1 |
| *S*. *cereale ssp. ancestrale* | | | Japan, Russia, USA | 2 | 18 |
| *S*. *cereale ssp. cereale* | | | Canada, Pakistan, Tajikistan |  |  |
| *S*. *cereale ssp. dighoricum* | | | Sweden |  |  |
| *S*. *cereale ssp. rigidum* | | | Poland |  |  |
| *S*. *cereale ssp. segetale* | | | Azerbaijan |  |  |
| *S*. *strictum ssp. africanum* | | | South Africa- RSA1 |  |  |
| *S*. *strictum ssp. anatolicum* | | | USA, Canada, Turkey |  |  |
| *S*. *strictum ssp. ciliatoglume* | | | Poland |  |  |
| *S*. *strictum spp. strictum* | | | Poland |  |  |
| *S*. *vavilovii* | | | Afghanistan, Russia, Poland |  |  |
| *S*. *cereale ssp. ancestrale* | | | Turkey | 3 | 1 |
| *S*. *cereale ssp. cereale* | | | USA | 4 | 3 |
| *S. strictum ssp*. *strictum* | | | Iraq |  |  |
| *S*. *vavilovii* | | | Hungary |  |  |
| *S*. *cereale ssp. segetale* | | | Russia | 5 | 1 |
| *S*. *cereal ssp. segetale* | | | Turkey | 6 | 1 |
| *S*. *cereal ssp. segetale* | | | Kazakhstan | 7 | 1 |
| *S*. *cereale spp. dighoricum* | | | Russia | 8 | 1 |
| *S*. *cereale spp. rigidum* | | | Turkey | 9 | 1 |
| *S*. *strictum ssp. africanum* | | | South Africa- RSA | 10 | 1 |
| *S*. *strictum ssp*. *strictum* | | | Hungary | 11 | 2 |
| *S*. *strictum ssp. ciliatoglume* | | | Poland |  |  |
| *S*. *strictum ssp. kuprijanovii* | | | Poland | 12 | 1 |
| *S*. *sylvestre* | | | Bulgaria | 13 | 1 |
| *S*. *sylvestre* | | | Russia | 14 | 1 |
| *S*. *sylvestre* | | | Poland | 15 | 1 |
| *S*. *sylvestre* | | | Hungary | 16 | 1 |
| **Total No. of Haplotype** | | | | | **35** |
