# Supplementary material for: Genetic diversity and relationship between cultivated, weedy and wild rye species as revealed by chloroplast and mitochondrial DNA non-coding regions analysis
Source: PLoS One. 2019 Feb 27;14(2):e0213023. doi: 10.1371/journal.pone.0213023 (PMC6392296; doi:10.1371/journal.pone.0213023)
Supplement: S9 Table — (DOCX) [file pone.0213023.s009.docx]

| **nad4/1-2** | | **Type of Haplotype** | **No. of Haplotype** |
| --- | --- | --- | --- |
| **Species /Subspcecies** | **Country of origin** |  |  |
| *S*. *cereale ssp. afghanicum* | Armenia | 1 | 1 |
| *S*. *cereale ssp. ancestrale* | Japan | 2 | 1 |
| *S*. *cereale ssp. ancestrale* | Russia | 3 | 9 |
| *S*. *cereale ssp. cereale* | Canada, Pakistan, USA, Tajikistan |  |  |
| *S*. *cereale ssp. dighoricum* | Sweden, Russia |  |  |
| *S*. *cereale ssp. rigidum* | Turkey, Poland |  |  |
| *S*. *cereale ssp. ancestrale* | Turkey | 4 | 1 |
| *S*. *cereale ssp. ancestrale* | USA | 5 | 1 |
| *S*. *cereal ssp. segetale* | Azerbaijan, Russia, Kazakhstan, Turkey | 6 | 8 |
| *S*. *sylvestre* | Russia, Hungary, Poland, Bulgaria |  |  |
| *S*. *strictum ssp. africanum* | South Africa- RSA, South Africa- RSA1 | 7 | 4 |
| *S*. *strictum ssp*. *strictum* | Hungary, Iraq |  |  |
| *S*. *strictum ssp. anatolicum* | USA, Canada | 8 | 4 |
| *S*. *strictum ssp. ciliatoglume* | Poland |  |  |
| *S*. *strictum ssp. kuprijanovii* | Poland |  |  |
| *S*. *strictum ssp. anatolicum* | Turkey | 9 | 1 |
| *S*. *strictum ssp*. *strictum* | Poland | 10 | 1 |
| *S. vavilowvii* | Afghanistan, Russia, Hungary | 11 | 3 |
| *S. vavilowvii* | Poland | 12 | 1 |
| **Total No. of Haplotype** | | | **35** |
| **rps12-nad3** | | **Type of Haplotype** | **No. of Haplotype** |
| **Species /Subspcecies** | **Country of origin** | 1 | 28 |
| *S*. *cereale ssp. afghanicum* | Armenia |  |  |
| *S*. *cereale ssp. ancestrale* | Japan, Russia |  |  |
| *S*. *cereale ssp. cereale* | USA, Tajikistan |  |  |
| *S*. *cereale ssp. dighoricum* | Sweden, Russia |  |  |
| *S*. *cereale ssp. rigidum* | Turkey, Poland |  |  |
| *S*. *cereal ssp. segetale* | Azerbaijan, Russia, Kazakhstan, Turkey |  |  |
| *S*. *strictum ssp. africanum* | South Africa- RSA, South Africa- RSA1 |  |  |
| *S*. *strictum ssp. anatolicum* | Turkey |  |  |
| *S*. *strictum ssp. ciliatoglume* | Poland |  |  |
| *S*. *strictum ssp. kuprijanovii* | Poland |  |  |
| *S*. *strictum ssp*. *strictum* | Poland, Hungary, Iraq |  |  |
| *S*. *sylvestre* | Russia, Hungary, Bulgaria,Poland |  |  |
| *S*. *cereale ssp. ancestrale* | Turkey, USA | 2 | 7 |
| *S*. *cereale ssp. cereale* | Canada, Pakistan |  |  |
| *S*. *strictum ssp. anatolicum* | Canada, USA |  |  |
| *S. vavilovii* | Afghanistan |  |  |
| **Total No. of Haplotype** |  |  | 35 |
